# Electronic and optical properties of lead-free hybrid double perovskites for photovoltaic and optoelectronic applications

**DOI:** 10.1038/s41598-018-37132-2

**Published:** 2019-01-24

**Authors:** Md Roknuzzaman, Chunmei Zhang, Kostya (Ken) Ostrikov, Aijun Du, Hongxia Wang, Lianzhou Wang, Tuquabo Tesfamichael

**Affiliations:** 1 School of Chemistry, Physics and Mechanical Engineering and Institute of Future Environments, Queensland University of Technology (QUT), Brisbane, QLD 4000 Australia; 2 School of Chemical Engineering and Australian Institute for Bioengineering and Nanotechnology, The University of Queensland, Brisbane, QLD 4072 Australia

## Abstract

Developing of lead-free double perovskites have drawn significant interest for photovoltaics and optoelectronics as the materials have the potential to avoid toxicity and instability issues associated with lead-based organometallic perovskites. In this study, we report the optoelectronic properties of a new group of non-toxic lead-free organic-inorganic halide double perovskites composed of caesium (Cs), methylammonium (MA) or formamidinium (FA) with bismuth (Bi) and metal copper (Cu). We perform density functional theory investigations to calculate the structural, electronic and optical properties of 18 Pb-free compounds, ABiCuX_6_ [A = Cs_2_, (MA)_2_, (FA)_2_, CsMA, CsFA, MAFA; X = I, Br, Cl] to predict their suitability in photovoltaic and optoelectronic applications. We found that the considered compounds are semiconductors with a tunable band gap characteristics that are suitable for some devices like light emitting diodes. In addition to this, the high dielectric constant, high absorption, high optical conductivity and low reflectivity suggest that the materials have the potential in a wide range of optoelectronic applications including solar cells. Furthermore, we predict that the organic-inorganic hybrid double perovskite (FA)_2_BiCuI_6_ is the best candidate in photovoltaic and optoelectronic applications as the material has superior optical and electronic properties.

## Introduction

Organic-inorganic hybrid perovskites having the general formula ABX_3_^1^, where A is a relatively large inorganic or organic cation (*e*.*g*., Cs^+^ or CH_3_NH_3_^+^), B is a metal cation (*e*.*g*., Pb^2+^) and X is a halogen anion (*e*.*g*., I^−^, Br^−^ or Cl^−^)^2^, is an emerging class of materials which have attracted significant attention in recent years because of their extraordinary optoelectronic characteristics such as tunable electronic bandgap, high optical absorption with broad spectrum, good photoconductivity, low carrier effective masses with high mobility, extended charge diffusion lengths with high lifetimes^3–9^. The materials have become more popular in the field of photovoltaic research because of its fastest growing Power Conversion Efficiency (PCE) as the PCE of the materials have been increased from 3.8%^1^ in 2009 to 22.7%^10^ in 2017. Their merits also enable the materials to be potentially useful in a variety types of optical and electronic devices beyond solar cells^6–9^. However, the materials still faces a huge challenge in large scale commercialization because of the structural instability against moisture/air and temperature as well as the toxicity of lead (Pb)^11,12^. Therefore, there is a great demand to find stable and nontoxic perovskites for the further development of perovskites solar cells. The insatiability issue of the halide perovskites can be treated by carbon encapsulation, multi-cation substitution as well as incorporation of hydrophobic moieties^13^, however the only way to address the toxicity of perovskite materials is the substitution of Pb by non-toxic elements^11^.

The most possible replacement of Pb are the elements which are in the same group in the periodic table *i*.*e*., those similar elements of group-IVA such as tin (Sn) and germanium (Ge). However, it has been reported that the Sn-based Pb-free perovskites are much less stable than Pb-containing ones because of the 2+ oxidation state of Sn, leading to a rapid degradation of the perovskite in air^14^. Also, the replacement of Pb by Ge results in a reduction of light absorption, dielectric constant and optical conductivity, leading to a decrease of the photovoltaic performance^9^. Similarly, lead-free perovskite materials containing other divalent elements except group-IVA have also been reported to have poor optoelectronic properties like high band gap, low absorption and large carrier effective masses, and consequently poor photovoltaic performance^11^. Clearly, the process of development of successful substitution of Pb for photovoltaic and optoelectronic applications is not trivial.

On the other hand, a complex substitution of Pb has been addressed by a combination of a trivalent and monovalent cations to form a new structure known as double perovskites which can be represented by the general formula, A_2_B′B″X_6_, where A is a relatively large cation (typically Cs^+^), B′ and B″ are either trivalent or monovalent cations, and X is either oxygen or halogen^2^. Recently, halide double perovskites have become more popular in the community of photovoltaic research because of its potential to overcome the instability and toxicity issues of Pb-based hybrid perovskites^2^. It is expected that studying of high number of possible combination of B′ and B″ in A_2_B′B″X_6_ can provide the opportunities to find effective alternatives of Pb-based halide perovskites^11^. The synthesis and characterization of a number of halide inorganic double perovskites (*e*.*g*., Cs_2_BiAgI_6_, Cs_2_BiAgBr_6_, Cs_2_BiAgCl_6_, Cs_2_InAgCl_6_)^15–19^ as well as halide hybrid double perovskites (*e*.*g*., MA_2_BiAgBr_6_, MA_2_BiKCl_6_, MA_2_BiTlBr_6_, MA_2_KGdCl_6_, MA_2_KYCl_6_)^20–23^ have been reported previously. However, most of the synthesized compounds have either large band gap values or poor optical properties, therefore the materials are not suitable for photovoltaic applications^24,25^. Recently, Volonakis *et*. *al*.^16^ have reported the optoelectronic properties of a group of halide double perovskites by heterovalent substitution of noble metals. Among the considered compounds, the double perovskite Cs_2_BiCuI_6_ has quite similar electronic properties like the most popular hybrid perovskite MAPbI_3_^9,16^. In addition to this, better optoelectronic properties have been observed for methylammonium (MA) or formamidinium (FA) containing organic-inorganic hybrid perovskite than Cs containing inorganic perovskites^8,9^. Herein, we introduce a new group of materials called organic-inorganic hybrid double perovskites by replacing Cs with a combination of Cs, MA and FA in inorganic Cs_2_BiCuX_6_ (X = I, Br, Cl). We calculate the structural, electronic and optical properties of 18 organic-inorganic double perovskites ABiCuX_6_ [A = Cs_2_, (MA)_2_, (FA)_2_, CsMA, CsFA, MAFA; X = I, Br, Cl] by first-principles density functional theory (DFT) simulations to find effective replacements of Pb-based perovskites for photovoltaic and optoelectronic applications.

## Results and Discussion

### Structural properties

**Figure 1:**
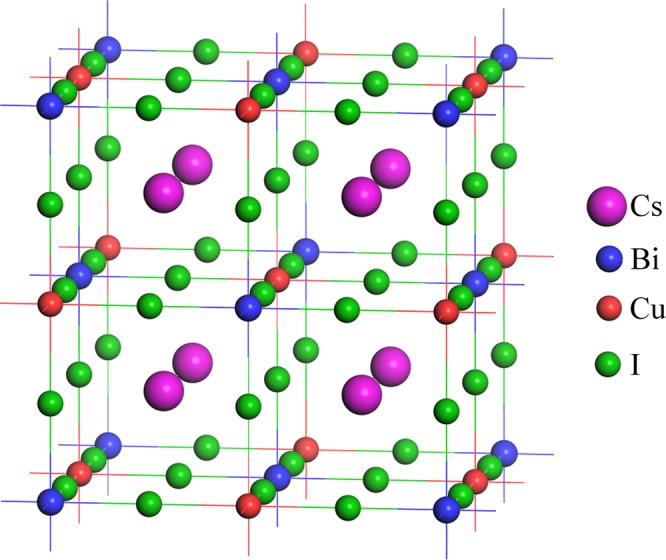
Unit cell of double perovskites Cs2BiCuI6 as an example of the crystal structure of the considered double perovskites ABiCuX6 [A = Cs2, (MA)2, (FA)2, CsMA, CsFA, MAFA; X = I, Br, Cl].

Double perovskites crystallize in cubic structure with space group ${Fm}\bar{3}m$ (no. 225) and can be represented by a general formula A_2_B′B″X_6_, where A atoms occupy the 8*c* Wyckoff position, B′ atoms occupy the 4*a* Wyckoff position, B″ atoms occupy the 4*b* Wyckoff position and X atoms occupy the 24*e* Wyckoff position with the fractional coordinates of (0.25, 0.25, 0.25), (0, 0, 0), (0.5, 0.5, 0.5) and (*x*, 0, 0), respectively^15^. Initially, the structures of Cs-based inorganic double perovskites have been drawn, then the structures are converted into 1 × 1 × 1 supercell to get the structures of organic-inorganic hybrid double perovskites. The Cs-based inorganic double perovskites have 40 atoms in their unit cell with four formula unit as shown in Fig. 1. After creating supercell, the required Cs atoms have been successfully replaced by organic MA or FA to obtain the structures of the desired organic-inorganic hybrid double perovskites (see Supplementary Fig. 1). The geometry optimization have been successfully performed for each model structure of the considered compounds using DFT simulations, as described in the Computational Methods Section. Also, the selected compounds are expected to be stable under considerations (see Supplementary document).

### Electronic properties

**Figure 2:**
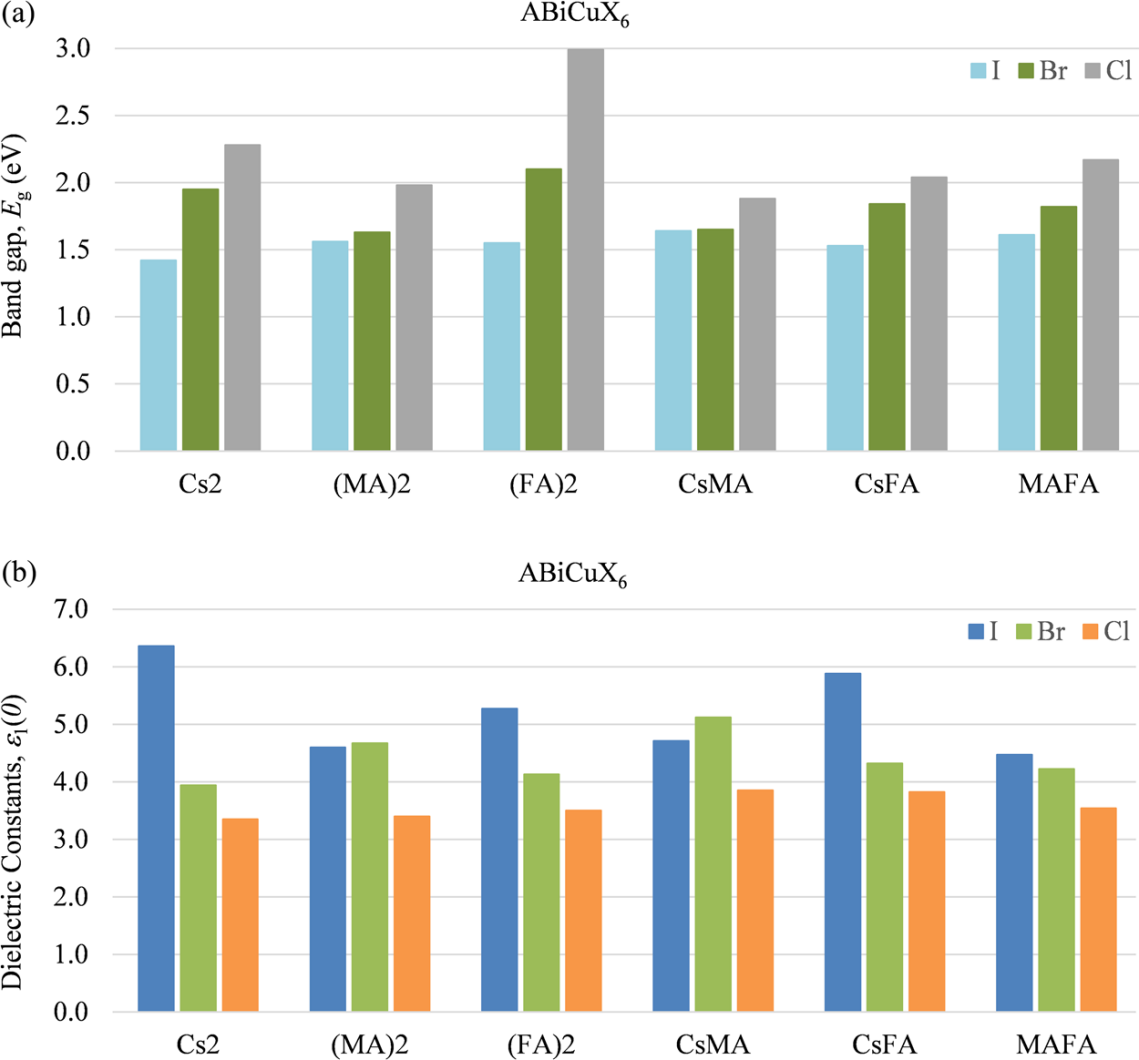
Comparison of the electronic band gap and dielectric constant of the selected group of double perovskites ABiCuX_6_ [A = Cs_2_, (MA)_2_, (FA)_2_, CsMA, CsFA, MAFA; X = I, Br, Cl]. (**a**) Electronic band gap of the materials determined by using GGA-PBE approach. (**b**) Calculated dielectric constant or static dielectric function for the considered double perovskites.

We investigate the electronic properties of the considered hypothetical organic-inorganic halide double perovskites using first-principles density functional theory (DFT)^26,27^ with the GGA-PBE approach^28^, executed in the CASTEP^29^ code of Materials Studio 2017, as explained in the Computational Methods Section. We further investigate the band structure of the considered compounds using hybrid HSE06^30^ functional as the usual GGA-PBE approach sometimes underestimate the band gap (see Supplementary Table 1). The bar chart of Fig. 2a shows the variation of the investigated band gap of the selected 18 double perovskites ABiCuX_6_ [A = Cs_2_, (MA)_2_, (FA)_2_, CsMA, CsFA, MAFA; X = I, Br, Cl]. Our results suggest that the replacement of Cs atoms by organic MA or FA has no significant effect on the band gap of the considered compounds. On the other hand, the variation of the band gap significantly depends on the substitution of halogen atoms. The band gap is observed to increase upon the substitution of I with Br and/or Cl atoms. This characteristic is also common in other group of materials like inorganic and organic-inorganic hybrid perovskites^8,9^. Therefore, the band gap of the considered compounds can be tuned by changing the halogen contents. This broad tunable band gap characteristic which covers the whole region of visible light spectra implies that the double perovskite compounds would be useful in areas such as light emitting diodes (LEDs). More specifically, the band gap varies more dramatically for the materials (FA)_2_BiCuX_6_ (X = I, Br, Cl) compared to other considered materials, suggesting that the double perovskites (FA)_2_BiCuX_6_ is the most suitable one to obtain the desired optimum band gap value for specific applications (*e*.*g*., solar cells, LEDs, laser) through bandgap engineering.

One of the major limitations of hybrid perovskites in solar cell application is that the materials have low dielectric constant because a low dielectric constant increases the rate of charge carrier recombination that in turn affect the overall device performance^31^. On the other hand, the materials with large dielectric constant can hold large quantities of charge for longer periods of time which enhance the photovoltaic performance. Therefore, it is important to find semiconductors with high dielectric constant. Our study suggests that few of our considered compounds (Cs_2_BiCuI_6_ and CsFABiCuI_6_) have higher dielectric constant than that of MAPbI_3_^9^. Among the considered compounds in this work, the highest dielectric constant of 6.36 is observed for Cs_2_BiCuI_6_. Moreover, the dielectric constant of (FA)_2_BiCuI_6_ is found to be 5.27 which is quite similar with the dielectric constant of MAPbI_3_ (5.23)^9^. Therefore, it is expected that the other optical properties of the double perovskite (FA)_2_BiCuI_6_ should be similar to the hybrid perovskite MAPbI_3_. On the other hand, as shown in Fig. 2b, the variation of the dielectric constant is almost inverse to the variation of the band gap for the 18 double perovskites considered. The dielectric constant decreases because of the substitution of I with other halogen atoms such as Br and Cl. However, the substitution of Cs by organic MA or FA has no effect on dielectric constant.

**Figure 3:**
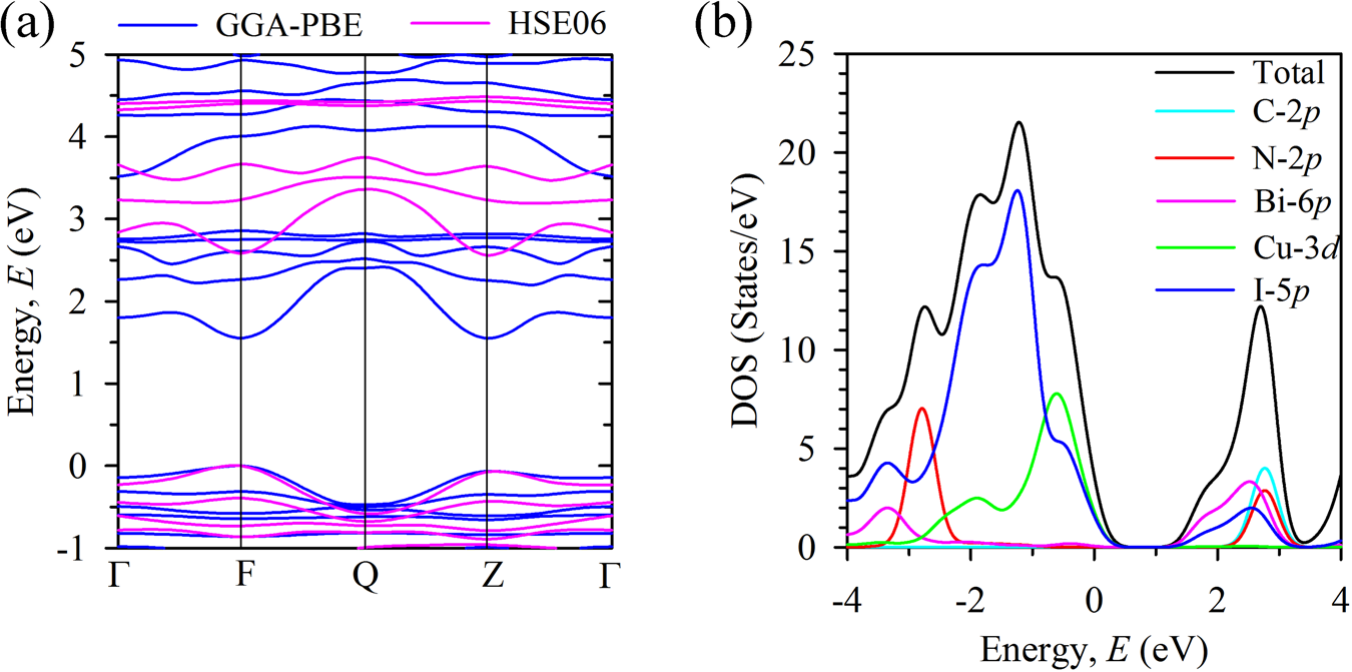
Calculated electronic properties of the considered double perovskite (FA)_2_BiCuI_6_. (**a**) Electronic band structure along the high symmetry direction of the Brillouin zone having path Γ(0,0,0)−F(0,0.5,0)−Q(0,0.5,0.5)−Z(0,0,0.5)− Γ(0,0,0). The bands calculated by GGA-PBE are indicated by blue color whereas the bands calculated by HSE06 are indicated by pink color. The valence band maximum (VBM) is seen at F point whereas the conduction band minimum (CBM) is observed at Z point of the Brillouin zone indicating that it is an indirect band gap semiconductor. (**b**) Calculated total and partial densities of states. The Cu-3*d* states (green color curve) and the I-5*p* (blue color curve) states are seen as the main contributors towards VBM whereas the Bi-6*p* (pink color curve) and I-5*p* (blue color curve) states are mostly contributed towards CBM.

Electronic band structure of a material reveal its type of band gap whereas the density of states (DOS) explore the contribution of atomic orbitals towards electronic states at VBM (valence band maximum) and CBM (conduction band minimum). We calculate the electronic band structure as well as the total and partial DOS of the 18 double perovskite compounds and the results are presented in the Supplementary Information (see Supplementary Figs 2, 3, 4 and 5). As our considered organic-inorganic double perovskite (FA)_2_BiCuI_6_ have similar dielectric constant to the most popular hybrid perovskite MAPbI_3_, therefore we further investigate its electronic properties (shown in Fig. 3) in details. The calculated results of the band structure (Fig. 3a) implies that the double perovskite (FA)_2_BiCuI_6_ is an indirect band gap semiconductor with the minimum band gap value of 1.55 eV for GGA-PBE and 2.56 eV for HSE06 (see Supplementary Table 1). In addition to this, it can be observed from Fig. 3a that the band structures derived for GGA-PBE and hybrid HSE06 have similar pattern, only the conduction band is seen to shift towards high energy for hybrid functional. On the other hand, the results of the total and partial DOS (Fig. 3b) indicate that the total DOS towards VBM is mainly contributed by Cu-3*d* states and I-5*p* states. However, significant contribution to the total DOS towards CBM mainly comes from Bi-6*p* and I-5*p* states. The halogen atoms are seen to contribute to the total DOS at both the VBM and CBM. This explains the observed phenomenon for the dramatic change of electronic band gap upon the change of halogen. The trend of the total and partial DOS is quite similar for the considered compounds (see Supplementary Figs 4 and 5) except FA containing compounds. An additional contribution to the total DOS at conduction band comes from C-2*p* and N-2*p* states for FA containing compounds. Also, the overlapping in partial DOS for C-2*p* and N-2*p* states suggesting that there is a hybridization between C-2*p* and N-2*p* orbitals.

### Optical properties

**Figure 4:**
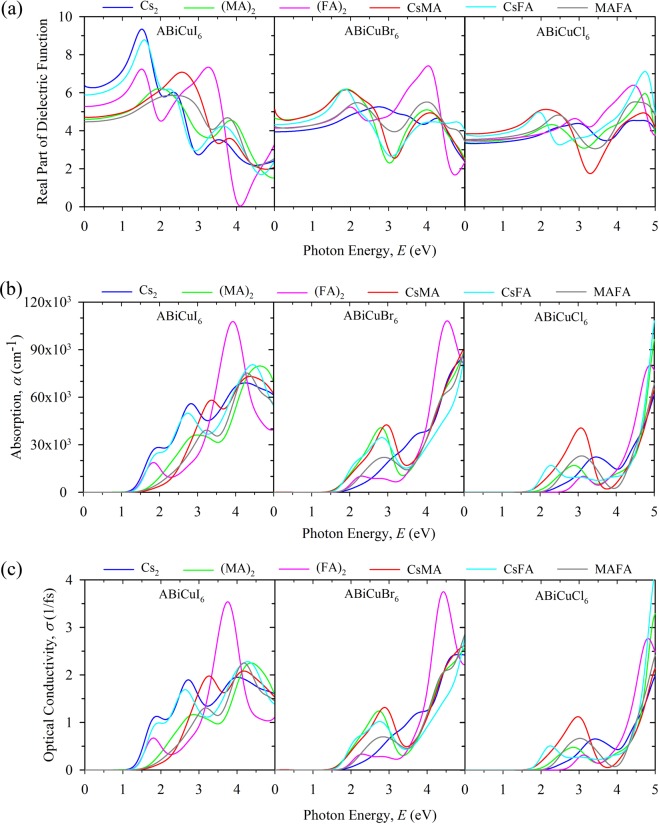
Comparison of the optical properties of double perovskites ABiCuX_6_ [A = Cs_2_, (MA)_2_, (FA)_2_, CsMA, CsFA, MAFA; X = I, Br, Cl] along the incident electromagnetic radiation of energy from 0 to 5 eV. (**a**) Calculated dielectric function (real part). (**b**) Calculated absorption coefficient. (**c**) Calculated optical conductivity.

We investigate the optical properties including dielectric function, optical absorption and conductivity, reflectivity, refractive index as well as the extinction coefficient for the considered double perovskites for the photon energy from 0 to 5 eV and the details of the calculations are presented in Supplementary Information. The calculated real part of the dielectric function for the considered compounds is shown in Fig. 4a. The results suggest that the materials Cs_2_BiCuI_6_ and (CsFA)BiCuI_6_ have higher dielectric constant at low energy region (from 0 to 2 eV). However, the double perovskite (FA)_2_BiCuI_6_ has maximum dielectric function at high energy region (from 2 to 4 eV) following a broad spectra compared to other compounds. Also, the material (FA)_2_BiCuI_6_ shows the highest value of imaginary dielectric function (Supplementary Fig. 6) compared to other compounds for solar radiation. Therefore, the high value of dielectric function of (FA)_2_BiCuI_6_ for solar radiation implies that it is a promising candidate for photovoltaic applications.

The absorption coefficient gives important knowledge on the light harvesting ability of a material, which affects the efficiency of solar energy conversion of corresponding solar cells as a consequence. Figure 4b presents the calculated absorption coefficient of the considered double perovskites. The materials show high absorption coefficient with a maximum absorption obtained with (FA)_2_BiCuI_6_. Therefore the Pb-free organic-inorganic hybrid double perovskite (FA)_2_BiCuI_6_ would be a potential alternative of Pb-based hybrid perovskites in solar cell application.

Furthermore, we investigate the optical conductivity of the materials. As shown in Fig. 4c, the variation of the optical conductivity of the double perovskites is similar for all compounds except (FA)_2_BiCuI_6_. The intense peak in the conductivity spectra of (FA)_2_BiCuI_6_ suggests it has the highest optical conductivity compared to others. The high value of optical conductivity also proves the superiority of (FA)_2_BiCuI_6_ for photovoltaic applications. For completeness, we also calculate the reflectivity, refractive index as well as the extinction coefficient for the considered materials (see Supplementary Figs 6 and 7). The low reflectivity (Supplementary Fig. 6b) and high extinction coefficient (Supplementary Fig. 7b) of (FA)_2_BiCuI_6_ are also evident that this double perovskite is the best candidate as a potential alternative of Pb-based halide hybrid perovskites for photovoltaic and optoelectronic applications such as solar cells and LEDs. In this study, we observe that the incorporation of organic component (especially FA) can enhance the photovoltaic and optoelectronic properties which may be because of the high conduction band DOS of FA containing compounds than its Cs containing counterparts.

## Conclusion

In summary, considering the properties of a potential 18 organic-inorganic lead-free double perovskites by changing Cs with different combinations of Cs, MA and FA in Cs_2_BiCuX_6_ (X = I, Br, Cl) based on first-principles DFT calculation, we have identified the organic-inorganic halide double perovskites (FA)_2_BiCuI_6_ is the most promising candidate which possess the desirable properties for photovoltaic and optoelectronic applications. The calculated band gap values suggest that the variation of band gap upon the replacement of halogen atoms is higher in (FA)_2_BiCuX_6_ (X = I, Br, Cl), which covers across the whole region of visible light spectra compared to that of other considered phases. This makes them ideal for colour light-emitting diodes. More specifically, the compound (FA)_2_BiCuI_6_ shows superior optoelectronic properties to the other compounds. It has high absorption coefficient, promising dielectric properties and high optical conductivity. Finally, the hybrid double perovskite (FA)_2_BiCuI_6_ has shown a promising results and would be a potential candidate to develop next generation Pb-free non-toxic materials for photovoltaics and optoelectronics.

### Computational Methods

The first-principles density functional theory (DFT)^26,27^ calculations have been performed by the plane wave pseudopotential method using the Cambridge Serial Total Energy Package (CASTEP)^29^ module of Materials Studio 2017. The geometry of the crystal was optimized using the Broyden–Fletcher–Goldfarb–Shanno (BFGS)^32^ minimization technique to find the ground state energy. The density mixing scheme^33^ was used to optimize the electronic structure. The generalized gradient approximation of Perdew-Berke-Ernzerhof (GGA-PBE)^28^ was used to evaluate the electronic exchange and correlation energy. The electronic exchange and correlation energy also treated by hybrid Heyd-Scuseria-Ernzerhof (HSE06)^30^ functional for the calculations of the band structure. The electron ion interaction was treated by Vanderbilt type ultrasoft pseudopotential^34^ for all calculations except for HSE06 functional in which norm conserving^35^ pseudopotential was used.

A plane-wave basis set of cutoff energy of 500 eV and the Monkhorst-Pack scheme^36^ *k*-points of 6 × 6 × 6 were used for all calculations associated with GGA-PBE as well as a cutoff energy of 800 eV and *k*-points of 2 × 2 × 2 were used for the rest calculations associated with HSE06. An acceptable convergence was affirmed by monitoring the cutoff energy and sampling of the Brillouin zone. The geometry of the crystals were successfully optimized with the convergence thresholds of 1 × 10^−5^ eV/atom for the total energy, 0.03 eV/Å for the maximum force, 0.05 GPa for the maximum stress and 1 × 10^−3^ Å for the maximum displacements.

## Supplementary information

Supplementary Document

## References

[1] Kojima A, Teshima K, Shirai Y, Miyasaka T (2009). Organometal Halide Perovskites as Visible-Light Sensitizers for Photovoltaic Cells. Journal of the American Chemical Society.

[2] Xiao Z (2017). Intrinsic Instability of Cs2In(I)M(III)X6 (M=Bi, Sb; X=Halogen) Double Perovskites: A Combined Density Functional Theory and Experimental Study. Journal of the American Chemical Society.

[3] Bakr OM, Mohammed OF (2017). Powering up perovskite photoresponse. Science.

[4] Yang Y, You J (2017). Make perovskite solar cells stable. Nature.

[5] Yin W-J, Shi T, Yan Y (2014). Unique Properties of Halide Perovskites as Possible Origins of the Superior Solar Cell Performance. Advanced Materials.

[6] Kovalenko MV, Protesescu L, Bodnarchuk MI (2017). Properties and potential optoelectronic applications of lead halide perovskite nanocrystals. Science.

[7] Chen J, Zhou S, Jin S, Li H, Zhai T (2016). Crystal organometal halide perovskites with promising optoelectronic applications. Journal of Materials Chemistry C.

[8] Roknuzzaman M, Ostrikov K, Wang H, Du A, Tesfamichael T (2017). Towards lead-free perovskite photovoltaics and optoelectronics by ab-initio simulations. Scientific Reports.

[9] Roknuzzaman M (2018). Insight into lead-free organic-inorganic hybrid perovskites for photovoltaics and optoelectronics: A first-principles study. Organic Electronics.

[10] NREL, https://www.nrel.gov/pv/assets/images/efficiency-chart.png (accessed 20/06/2018).

[11] Zhang P, Yang J, Wei S-H (2018). Manipulation of cation combinations and configurations of halide double perovskites for solar cell absorbers. Journal of Materials Chemistry A.

[12] Shahbazi M, Wang H (2016). Progress in research on the stability of organometal perovskite solar cells. Solar Energy.

[13] Tiing TV (2018). Octadecylamine-Functionalized Single-Walled Carbon Nanotubes for Facilitating the Formation of a Monolithic Perovskite Layer and Stable Solar Cells. Advanced Functional Materials.

[14] Noel NK (2014). Lead-free organic-inorganic tin halide perovskites for photovoltaic applications. Energy & Environmental Science.

[15] Filip MR, Hillman S, Haghighirad AA, Snaith HJ, Giustino F (2016). Band Gaps of the Lead-Free Halide Double Perovskites Cs2BiAgCl6 and Cs2BiAgBr6 from Theory and Experiment. The Journal of Physical Chemistry Letters.

[16] Volonakis G (2016). Lead-Free Halide Double Perovskites via Heterovalent Substitution of Noble Metals. The Journal of Physical Chemistry Letters.

[17] Slavney AH, Hu T, Lindenberg AM, Karunadasa HI, Bismuth-Halide Double A (2016). Perovskite with Long Carrier Recombination Lifetime for Photovoltaic Applications. Journal of the American Chemical Society.

[18] Volonakis G (2017). Cs2InAgCl6: A New Lead-Free Halide Double Perovskite with Direct Band Gap. The Journal of Physical Chemistry Letters.

[19] Yang B (2018). Lead-Free Silver-Bismuth Halide Double Perovskite Nanocrystals. Angewandte Chemie International Edition.

[20] Wei F (2016). The synthesis, structure and electronic properties of a lead-free hybrid inorganic-organic double perovskite (MA)2KBiCl6 (MA = methylammonium). Materials Horizons.

[21] Deng Z (2016). Exploring the properties of lead-free hybrid double perovskites using a combined computational-experimental approach. Journal of Materials Chemistry A.

[22] Wei F (2017). Synthesis and Properties of a Lead-Free Hybrid Double Perovskite: (CH3NH3)2AgBiBr6. Chemistry of Materials.

[23] Deng Z (2017). Synthesis and Characterization of the Rare-Earth Hybrid Double Perovskites: (CH3NH3)2KGdCl6 and (CH3NH3)2KYCl6. The Journal of Physical Chemistry Letters.

[24] Volonakis G, Haghighirad AA, Snaith HJ, Giustino F (2017). Route to Stable Lead-Free Double Perovskites with the Electronic Structure of CH3NH3PbI3: A Case for Mixed-Cation [Cs/CH3NH3/CH(NH2)2]2InBiBr6. The Journal of Physical Chemistry Letters.

[25] Meng W (2017). Parity-Forbidden Transitions and Their Impact on the Optical Absorption Properties of Lead-Free Metal Halide Perovskites and Double Perovskites. The Journal of Physical Chemistry Letters.

[26] Hohenberg P, Kohn W (1964). Inhomogeneous Electron Gas. Physical Review.

[27] Kohn W, Sham LJ (1965). Self-Consistent Equations Including Exchange and Correlation Effects. Physical Review.

[28] Perdew JP, Burke K, Ernzerhof M (1996). Generalized Gradient Approximation Made Simple. Physical Review Letters.

[29] Clark SJ (2005). First principles methods using CASTEP. Zeitschrift fur Kristallographie.

[30] Heyd J, Scuseria GE, Ernzerhof M (2003). Hybrid functionals based on a screened Coulomb potential. The Journal of Chemical Physics.

[31] Liu X (2018). A high dielectric constant non-fullerene acceptor for efficient bulk-heterojunction organic solar cells. Journal of Materials Chemistry A.

[32] Fischer TH, Almlof J (1992). General methods for geometry and wave function optimization. The Journal of Physical Chemistry.

[33] Kresse G, Furthmüller J (1996). Efficient iterative schemes for ab initio total-energy calculations using a plane-wave basis set. Physical Review B.

[34] Vanderbilt D (1990). Soft self-consistent pseudopotentials in a generalized eigenvalue formalism. Physical Review B.

[35] Kleinman L, Bylander DM (1982). Efficacious Form for Model Pseudopotentials. Physical Review Letters.

[36] Monkhorst HJ, Pack JD (1976). Special points for Brillouin-zone integrations. Physical review B.

