# Supplementary material for: Electronic and optical properties of lead-free hybrid double perovskites for photovoltaic and optoelectronic applications
Source: Sci Rep. 2019 Jan 24;9:718. doi: 10.1038/s41598-018-37132-2 (PMC6345881; doi:10.1038/s41598-018-37132-2)
Supplement: Supplementary file 1 — Supplementary Document [file 41598_2018_37132_MOESM1_ESM.pdf]

Supplementary Information:

Electronic and optical properties of lead-free hybrid double perovskites for photovoltaic and optoelectronic applications

Md Roknuzzaman[1], Chunmei Zhang[1], Kostya (Ken) Ostrikov[1], Aijun Du[1], Hongxia Wang[1], Lianzhou Wang[2], and Tuquabo Tesfamichael[1,*]

[1]School of Chemistry, Physics and Mechanical Engineering and Institute of Future Environments, Queensland University of Technology (QUT), QLD 4000, Australia

[2]School of Chemical Engineering and Australian Institute for Bioengineering and Nanotechnology, The University of Queensland, Brisbane, QLD, 4072, Australia

Calculations of Optical Properties: The complex dielectric function of a crystalline solid can be expressed by the equation, $\varepsilon(\omega) = \varepsilon_1(\omega) + i\varepsilon_2(\omega)$; where $\varepsilon_1(\omega)$ and $\varepsilon_2(\omega)$ are the real and imaginary parts of the dielectric function, respectively. The imaginary part of the dielectric function is required to evaluate other optical constants[1] and can be derived using the momentum matrix elements related to the occupied and unoccupied wave functions in accordance with the selection rules[2] and is expressed as:

$$\varepsilon_2(\omega) = \frac{2e^2\pi}{\Omega\varepsilon_0}\sum_{k,v,c}|\langle\psi_k^c|\boldsymbol{u}\cdot\boldsymbol{r}|\psi_k^v\rangle|^2\,\delta(E_k^c - E_k^v - E)$$

Here, ω is the phonon frequency, e is the electronic charge, Ω is the volume of a unit cell, $\boldsymbol{u}$ is the unit vector along the polarization of the incident electric field and ψ_k^c and ψ_k^v are respective wave functions for conduction and valence band electrons, respectively at a particular k. For the derivation of the real part of the dielectric function from imaginary part, the Kramers-Kronig relations[3] can be used. The remaining optical properties like absorption, optical conductivity, reflectivity, refractive index, extinction coefficient can be evaluated by using the real and imaginary parts of the dielectric function as reported in the literature[4].

* Corresponding author.
Email address: t.tesfamichael@qut.edu.au (T. Tesfamichael)

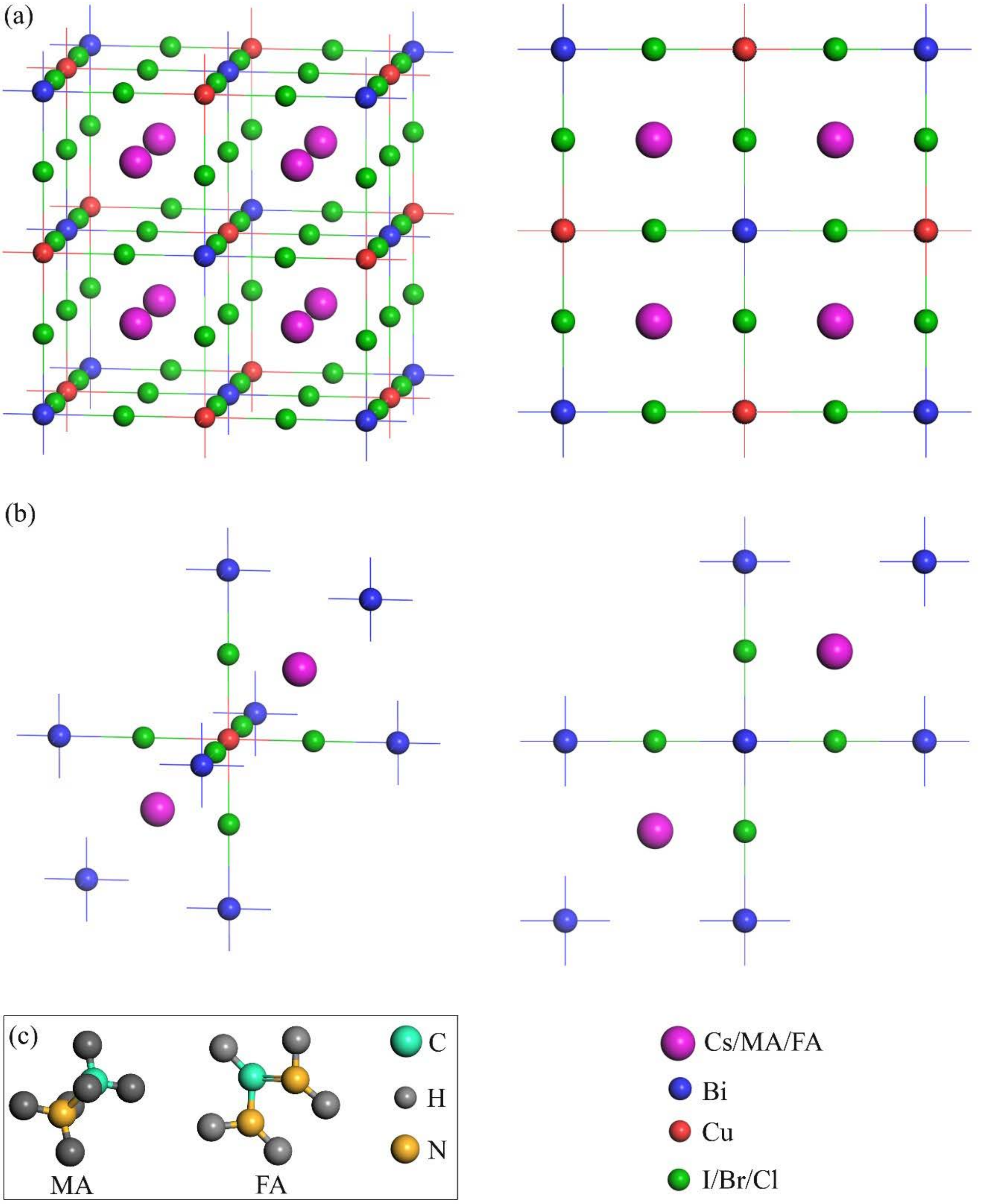

Supplementary Figure 1. Crystal structure of organic-inorganic double perovskites. The structure of inorganic double perovskite Cs_2BiCuI_6 was initially drawn. Then the Cs atoms were replaced by MA (methylammonium) or FA (formamidinium) to get the structure of required organic-inorganic double perovskites. (a) Three and two dimensional views of the unit cell of inorganic double perovskite Cs_2BiCuI_6. (b) Three and two dimensional views of the primitive cell of inorganic double perovskite Cs_2BiCuI_6. (c) Molecular structure of CH_3NH_3 (or, MA) and $CH(NH_2)_2$ (or, FA). The carbon-nitrogen double bond is also shown in the figure.

Supplementary Table 1. The calculated enthalpy of formation, *H* (KJ/mol), electronic band gap, E_g (eV) for GGA-PBE and hybrid HSE06 potential, and dielectric constants, $\varepsilon_1(0)$ of inorganic-organic double perovskites $ABiCuX_6$: [A = Cs_2, $(MA)_2$, $(FA)_2$, CsMA, CsFA, MAFA; X = I, Br, Cl].

Compounds	*H*	E_g		$\varepsilon_1(0)$	Compounds	*H*	E_g		$\varepsilon_1(0)$
		GGA-PBE	HSE06				GGA-PBE	HSE06	
Cs_2BiCuI_6	-342.6	1.42	2.42	6.36	$(CsMA)BiCuI_6$	-369.1	1.64	2.67	4.71
$Cs_2BiCuBr_6$	-467.6	1.95	2.78	3.94	$(CsMA)BiCuBr_6$	-515.9	1.65	2.84	5.12
$Cs_2BiCuCl_6$	-666.5	2.29	3.11	3.35	$(CsMA)BiCuCl_6$	-765.6	1.88	3.15	3.85
$(MA)_2BiCuI_6$	-400.4	1.56	2.65	4.60	$(CsFA)BiCuI_6$	-385.4	1.53	2.58	5.88
$(MA)_2BiCuBr_6$	-576.3	1.63	2.81	4.67	$(CsFA)BiCuBr_6$	-535.2	1.84	2.83	4.32
$(MA)_2BiCuCl_6$	-901.2	1.98	3.24	3.40	$(CsFA)BiCuCl_6$	-787.7	2.04	3.25	3.82
$(FA)_2BiCuI_6$	-434.9	1.55	2.56	5.27	$(MAFA)BiCuI_6$	-417.9	1.61	2.73	4.47
$(FA)_2BiCuBr_6$	-616.9	2.10	3.42	4.13	$(MAFA)BiCuBr_6$	-596.9	1.82	3.11	4.22
$(FA)_2BiCuCl_6$	-946.0	2.99	4.18	3.50	$(MAFA)BiCuCl_6$	-923.9	2.17	3.53	3.54

Stability: The enthalpy of formation of a material is the change of enthalpy during the formation of 1 mole of the compound from its constituent elements. A negative formation enthalpy of a compound suggests that the material is stable while a more negativity implies more stability[5]. The formation enthalpy of the considered compounds is presented in Supplementary Table 1. The enthalpy of FA containing compounds are found as more negative compared to its counterparts of other A site cation compounds suggesting the FA-based compounds are more stable than others. On the other hand, the calculated results suggest that the iodine-based compounds are less stable compared to its Br and Cl containing counterparts. However, the negative formation enthalpy of the considered compounds implies that the materials are expected to be stable under considerations.

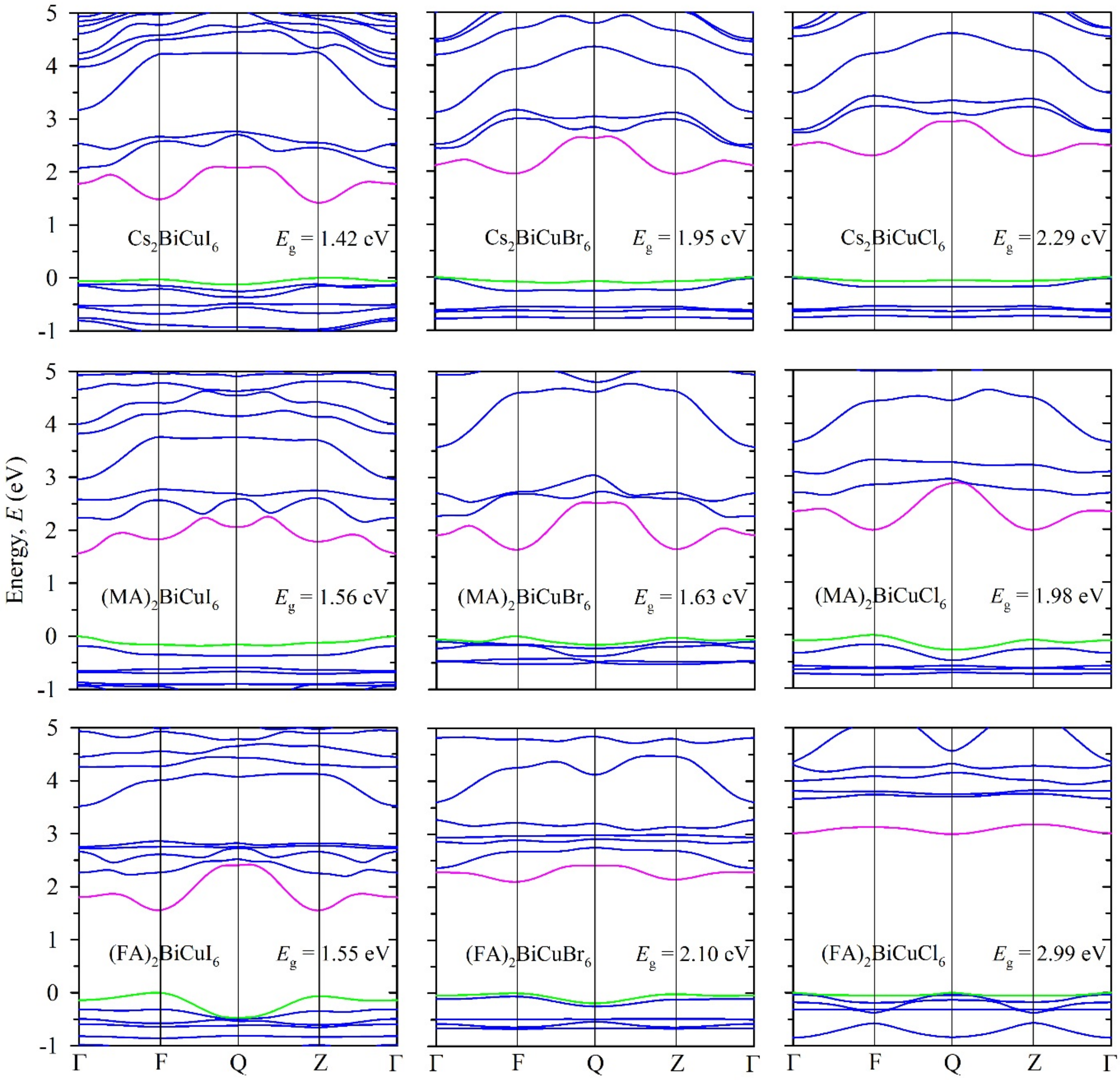

Supplementary Figure 2. Calculated electronic band structure of the considered double perovskites $ABiCuI_6$ [A = Cs_2, $(MA)_2$, $(FA)_2$; X = I, Br, Cl] along the high symmetry direction of the Brillouin zone having path Γ(0,0,0)–F(0,0.5,0)–Q(0,0.5,0.5)–Z(0,0,0.5)– Γ(0,0,0). The green band indicates the highest energy valence band in which the valence band maximum (VBM) is observed. On the other hand, the pink band indicates the lowest energy conduction band in which the conduction band minimum (CBM) is observed.

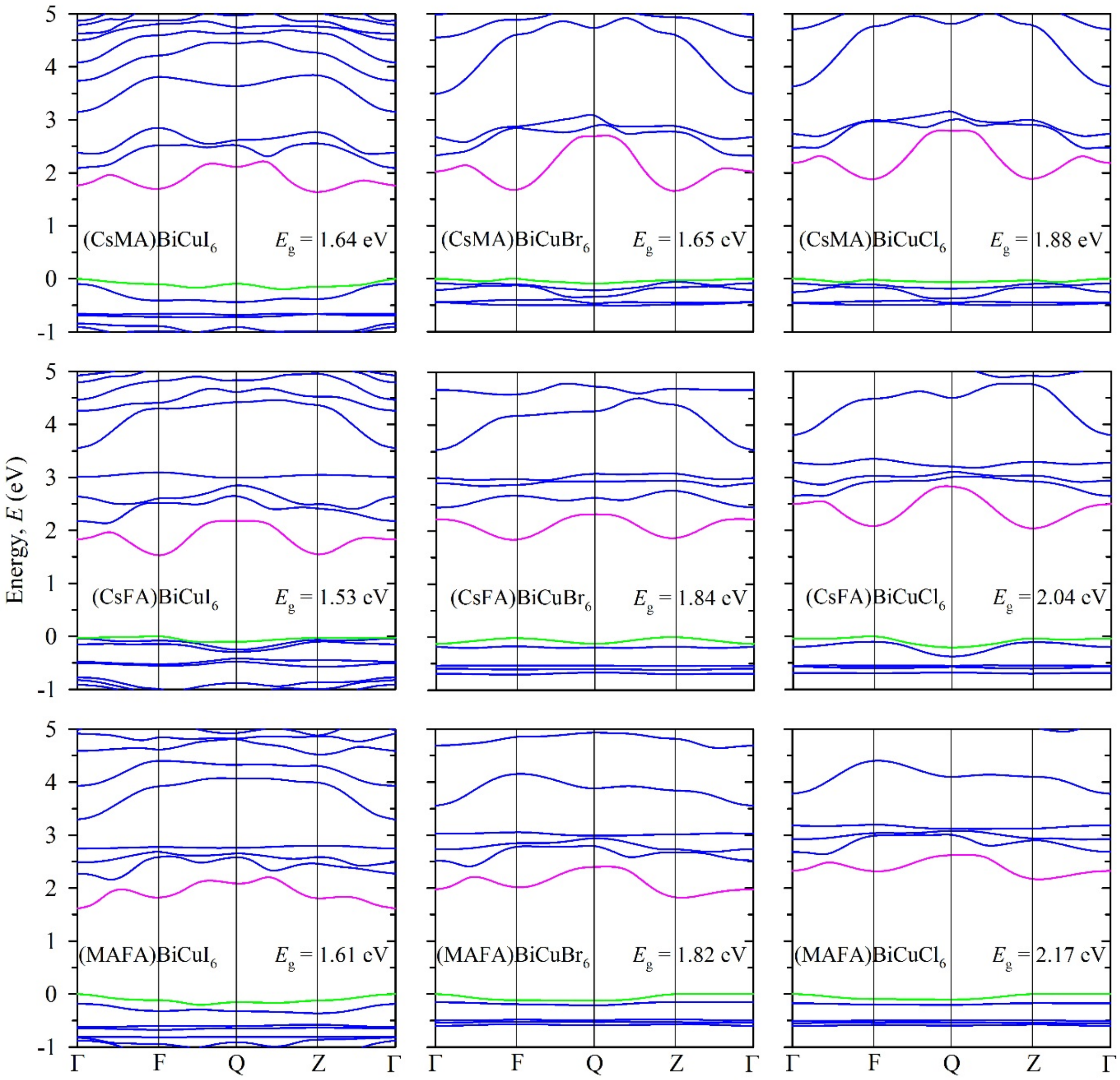

Supplementary Figure 3. Calculated electronic band structure of the considered double perovskites ABiCuI$_6$ [A = CsMA, CsFA, MAFA; X = I, Br, Cl] along the high symmetry direction of the Brillouin zone having path Γ(0,0,0)─F(0,0.5,0)─Q(0,0.5,0.5)─Z(0,0,0.5)─ Γ(0,0,0). The green band indicates the highest energy valence band in which the valence band maximum (VBM) is observed. On the other hand, the pink band indicates the lowest energy conduction band in which the conduction band minimum (CBM) is observed.

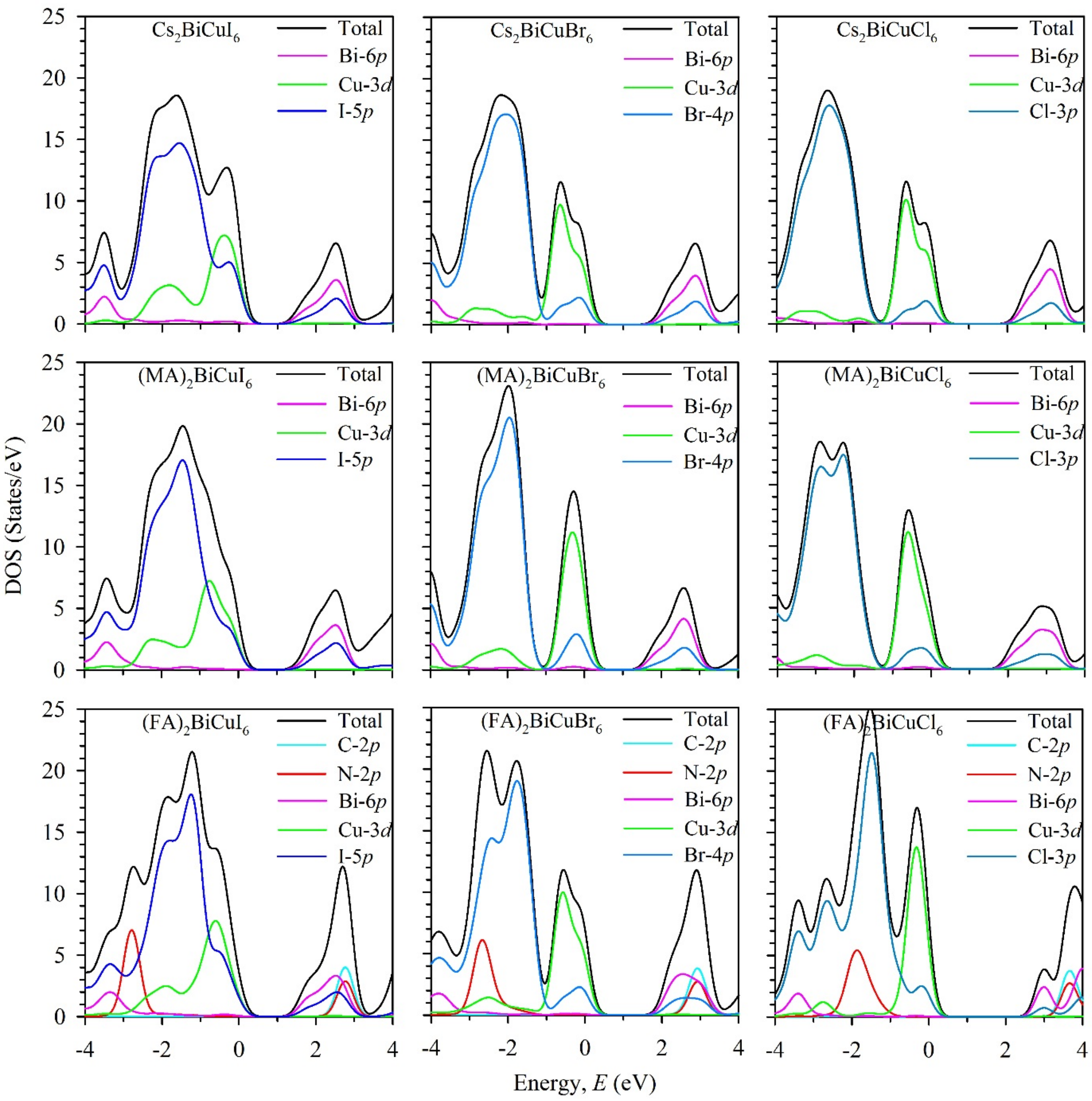

Supplementary Figure 4. Calculated total and partial densities of states of the considered double perovskites ABiCuI6 (A = Cs_2, $(MA)_2$, $(FA)_2$; X = I, Br, Cl). The Cu-3*d* states (blue colour curve) and the *p* states of halogen (I-5*p*, Br-4*p*, Cl-3*p*) are seen to contribute towards valence band maximum (VCM). The *p* states of halogen is also contributed to conduction band minimum (CBM). However, the maximum contribution towards the CBM is mainly come from Bi-6*p* states (pink colour curve).

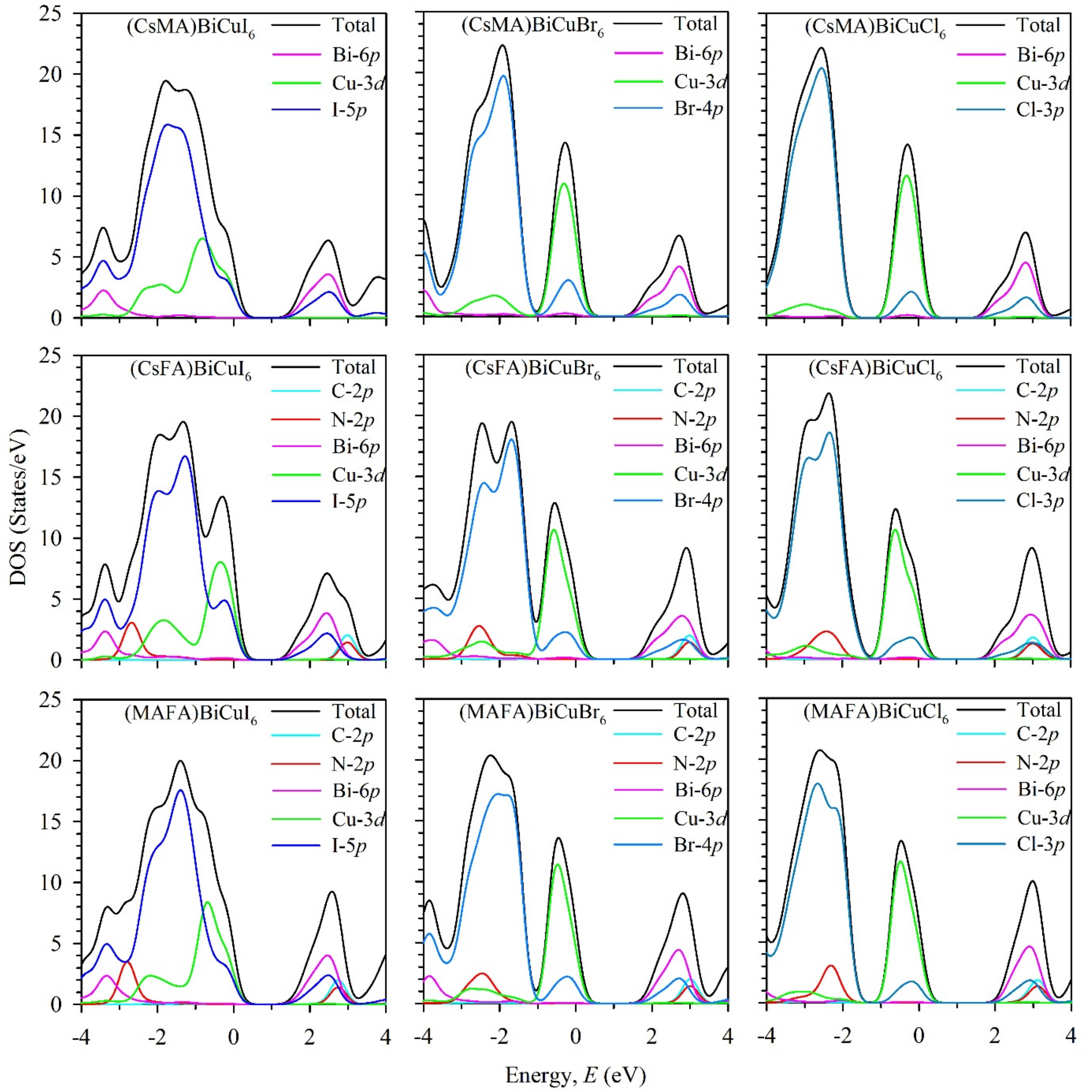

Supplementary Figure 5. Calculated total and partial densities of states of the considered double perovskites ABiCuI6 (A = CsMA, CsFA, MAFA; X = I, Br, Cl). The Cu-3*d* states (blue colour curve) and the *p* states of halogen (I-5*p*, Br-4*p*, Cl-3*p*) are seen to contribute towards valence band maximum (VCM). The *p* states of halogen are also contributed to conduction band minimum (CBM). However, the maximum contribution towards the CBM is mainly come from Bi-6*p* states (pink colour curve).

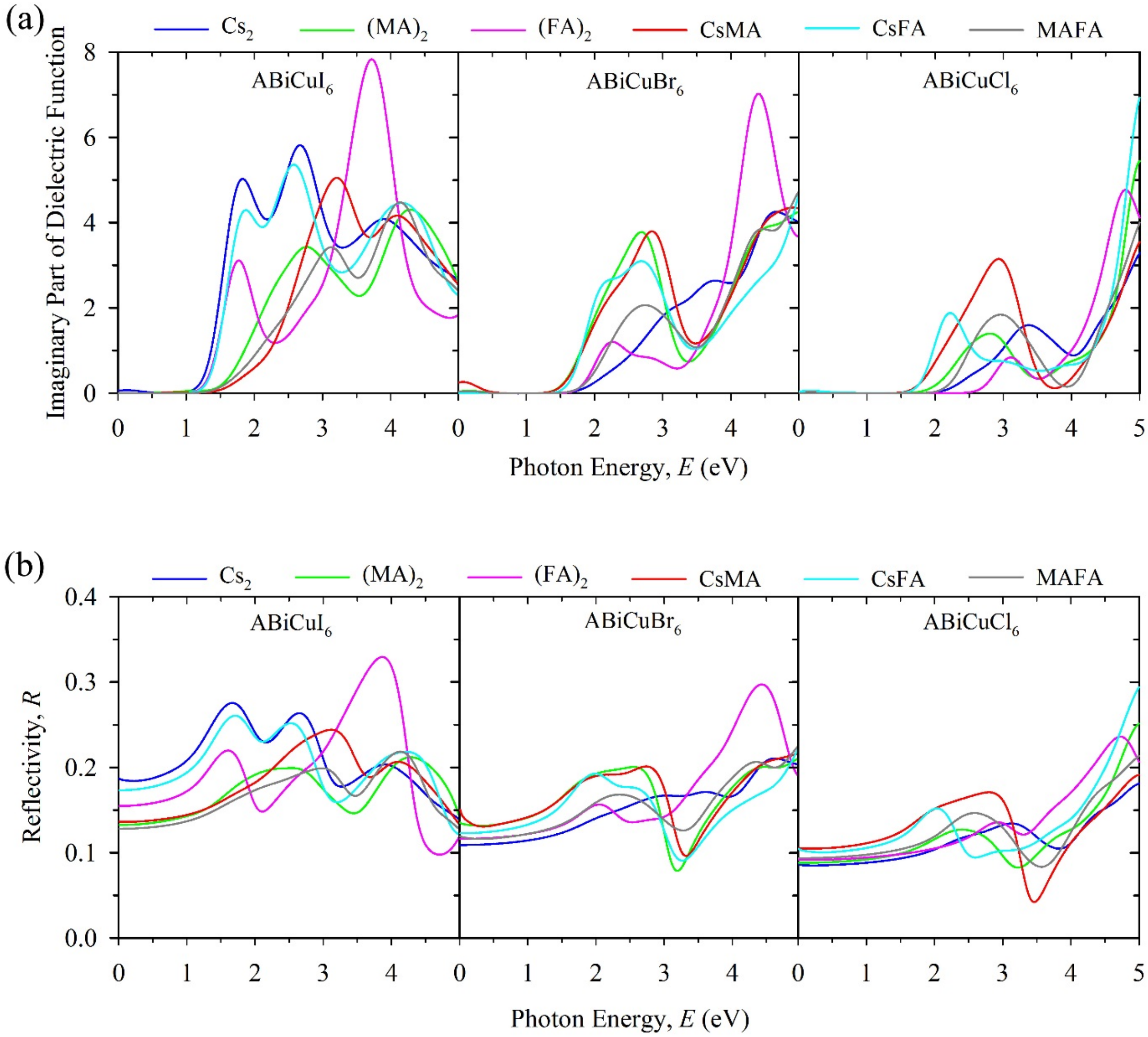

Supplementary Figure 6. Calculated (a) Imaginary part of dielectric function and (b) Reflectivity of the considered double perovskites $ABiCuI_6$ (A = A = Cs_2, $(MA)_2$, $(FA)_2$, CsMA, CsFA, MAFA; X = I, Br, Cl). The variation of the imaginary part of the dielectric function is quite similar for the considered compounds except an intense peak of $(FA)_2BiCuI_6$ and $(FA)_2BiCuI_6$ at the energy 3.75 eV and 4.3 eV, respectively. The reflectivity of the considered compounds is seen to vary between 12 to 30 % implies that the materials have low reflectivity for incoming solar radiation.

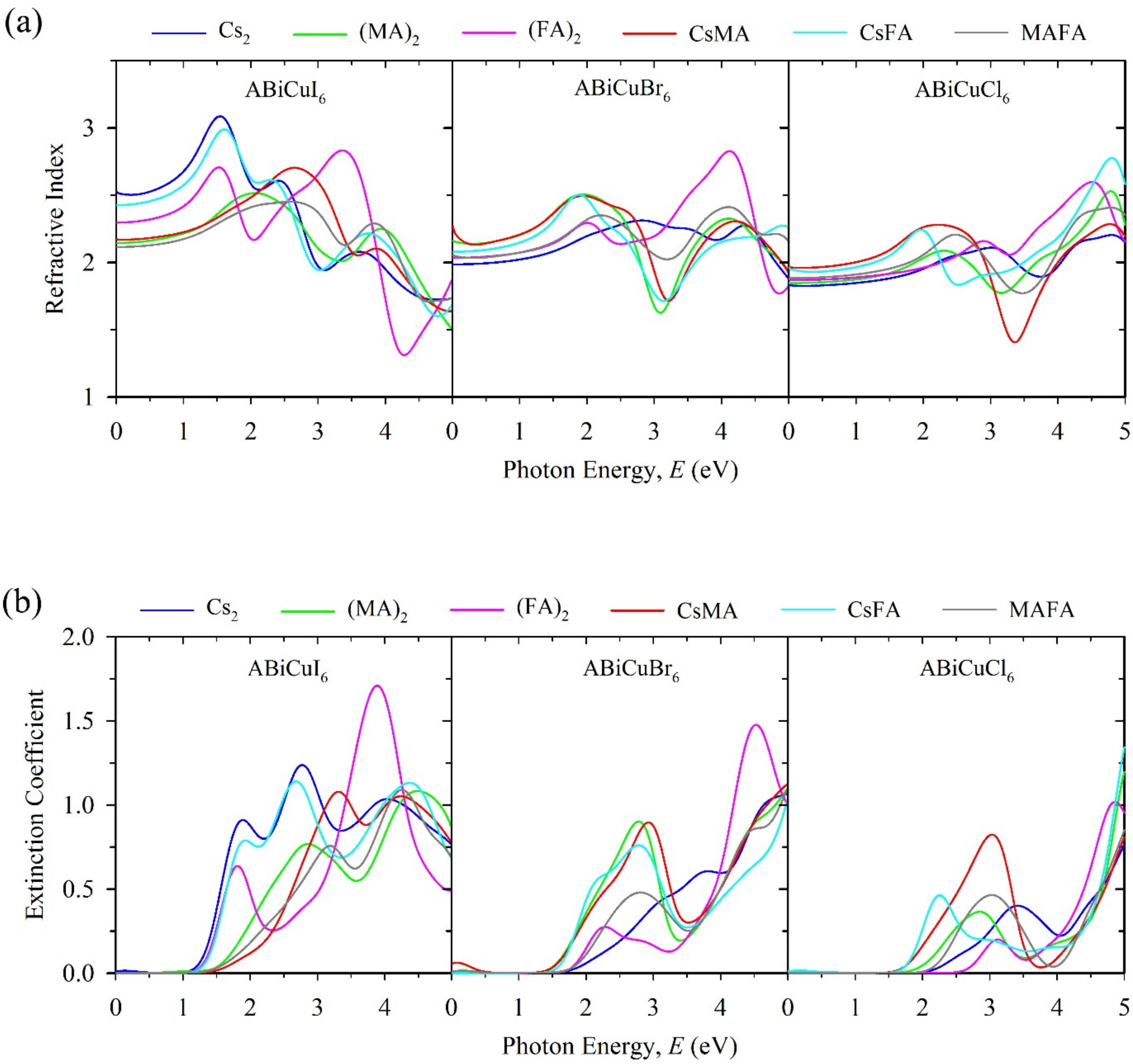

Supplementary Figure 7. Calculated (a) Refractive Index and (b) Extinction Coefficient of the considered double perovskites $ABiCuI_6$ (A = A = Cs_2, $(MA)_2$, $(FA)_2$, CsMA, CsFA, MAFA; X = I, Br, Cl). The variation of the refractive index is quite similar for the considered compounds. However, an intense peak is observed in the extinction coefficient spectrum of $(FA)_2BiCuI_6$ double perovskite, this suggests the potential of the material to be used in solar cell and other optoelectronic devices.

References

1. Roknuzzaman, M. *et al.* First hafnium-based MAX phase in the 312 family, Hf3AlC2: A first-principles study. *Journal of Alloys and Compounds* **727**, 616-626, doi:https://doi.org/10.1016/j.jallcom.2017.08.151 (2017).
2. Harris, D. C. & Bertolucci, M. D. *Symmetry and spectroscopy: an introduction to vibrational and electronic spectroscopy*. (Courier Corporation, 1978).
3. Grundmann, M. in *The Physics of Semiconductors: An Introduction Including Nanophysics and Applications* 775-776 (Springer Berlin Heidelberg, 2010).
4. Hadi, M. A., Vovk, R. V. & Chroneos, A. Physical properties of the recently discovered $Zr_2(Al_{1-x}Bi_x)C$ MAX phases. *Journal of Materials Science: Materials in Electronics* **27**, 11925-11933, doi:10.1007/s10854-016-5338-z (2016).
5. Roknuzzaman, M. *et al.* Physical properties of predicted Ti_2CdN versus existing Ti_2CdC MAX phase: An ab initio study. *Computational Materials Science* **113**, 148-153, doi:https://doi.org/10.1016/j.commatsci.2015.11.039 (2016).
